# K*_x_*WO Is a Novel Ferroelectric Nanomaterial for Application as a Room Temperature Acetone Sensor

**DOI:** 10.3390/nano10020225

**Published:** 2020-01-28

**Authors:** Michael E. Johnson, Qifeng Zhang, Danling Wang

**Affiliations:** 1 Materials and Nanotechnology Program, North Dakota State University, Fargo, ND 58102, USA; michael.johnson.1@ndsu.edu (M.E.J.); qifeng.zhang@ndsu.edu (Q.Z.); 2 Department of Electrical and Computer Engineering, North Dakota State University, Fargo, ND 58102, USA

**Keywords:** sensors, diabetes, ferroelectric nanomaterial, acetone sensor, chemiresistive response

## Abstract

A newly synthesized nanomaterial known as K*_x_*W_7_O_22_ (K*_x_*WO) exhibits a stable room-temperature ferroelectric property. This unique ferroelectric property has revealed that K*_x_*WO is a promising material for application in a breath sensor, which can be used for patients to monitor their daily health condition and diagnose disease at every early stage with low cost, convenience, and non-invasion. In this study, we successfully synthesized nano-structured K*_x_*WO through a low cost but high yield hydrothermal method. The sensing response of K*_x_*WO to acetone is examined based on a chemiresistive effect. For the first time, we systematically studied how material structures and the component, potassium (K), can affect K*_x_*WO-based sensing performance. The results indicate that the low temperature ferroelectric property of K*_x_*WO causes an excellent response to acetone, which is the biomarker for diabetes. The lowest detection limit can be down to 0.1 ppm and the K*_x_*WO-based sensor can operate at room temperature. In addition, the K*_x_* component K*_x_*WO and its crystal structure also play an important role in improving its sensing performance. Our results provide advanced research in (1) exploring the study of K*_x_*WO material properties by tailoring the concentration of the potassium in K*_x_*WO and introducing the surfactant Pluronic L-121 in the growing process, and (2) optimizing K*_x_*WO sensing performance by controlling its material properties.

## 1. Introduction

Diabetes is one of the leading causes of health complications and death in the United States. Currently, diagnosing and monitoring methods are invasive with risk of infection and are costly due to the requirement of regularly replacing either lancets, needles, or sensing strips. A breathalyzer for diabetes monitoring and diagnosis would have none of these downsides and would yield fast results that could also be collected for big data analysis. To be able to detect a disease via the breath, we need to identify a volatile-organic-compound (VOC) that functions as a biomarker for diabetes. Conveniently, it has been shown that acetone in the breath can be used as the biomarker for diabetes, and it directly correlates with blood-glucose concentrations [1]. By sensing acetone in the breath, we can perform a rapid and non-invasive testing method for screening and diagnosing diabetes [2].

Typically, the amount of acetone in a healthy person’s breath is around 0.76 ppm, and those with diabetes can have a breath-acetone-concentration of upwards of 1.71 ppm [3]. Currently, non-invasive methods for detecting VOCs have been developed by using gas chromatography/mass spectrometry and solid-phase microextraction. These techniques are highly effective and have low detection limits of 0.049 ppb [3]. However, these techniques require large bulky machinery that need highly skilled personnel to operate. A breathalyzer would eliminate the need for such bulky equipment and would have simpler user interface that anyone would be able to conveniently operate it in a clinical or non-clinical setting.

Tungsten oxide (WO_3_) is an *n*-type semiconductor, which has shown an excellent sensing application in detecting gases such as ethanol, acetone, NH_3_, formaldehyde, toluene, and H_2_S [4,5,6,7]. Additionally, tungsten oxide-based sensors are easy to fabricate, low-cost, and have great potential for miniaturization. Sensors designed using nanostructured WO_3_ are small, robust, inexpensive, and have high sensitivities, which makes them an excellent choice for handheld devices to be used in the medical field [4]. However, most semiconductor metal oxide sensors including WO_3_-based sensors have to operate at an elevated temperature ranging from 100–500 °C due to the ionosorption and oxygen vacancy sensing mechanism [4,5,6,7,8,9,10,11,12,13,14,15]. This type of sensor eventually results in a high-power consumption device and the thermal deterioration of sensing performance. In our previous research, we have successfully synthesized and fabricated an acetone sensor based on a novel material known as a K_2_W_7_O_22_ (KWO) nanorod [16]. KWO, as a *p*-type semiconducting nanomaterial, has shown to have many advantages over WO_3_ for sensing acetone including low operating temperature (~22 °C), excellent sensitivity, and long-term reliability. Compared to sensors operating at an elevated temperature, a KWO-based sensor can work at room-temperature, which would require much less power and complexity due to a heating element being unnecessary. Our previous results revealed KWO has a very different mechanism for sensing acetone. Therefore, the unique material property of KWO, which is a low-temperature ferroelectric property, plays an important role in acetone detection, which causes efficient charge transfer between acetone and KWO even at room temperature [16]. In this paper, we focus on the understanding the origin of the ferroelectric property in KWO as well as how the material’s structure and components can affect the ferroelectric property and then improve KWO’s response to acetone. 

Generally, exposed crystal facets and crystal structures of nanomaterials can determine the performance of gas sensors [14,17,18,19,20,21]. Research on WO_3_ has proved that manipulating the surface facets of WO_3_ nanomaterials can greatly impact their sensing properties [14]. The addition of surfactants during growth can influence the morphology and surface facets [17]. Motivated by this research, we have carried out research focusing on the specific surface facets of KWO using certain surfactants during growth and by controlling the ratio of potassium (K) in KWO. The preliminary results show that both the ratio of K and surfactants can influence KWO sensing performance. As mentioned earlier, KWO has a strong room-temperature ferroelectric property that drives a different mechanism for sensing acetone. This room-temperature ferroelectric property has been correlated to the ε-WO_3_ phase like ε-phase in WO_3_ [22]. Therefore, by manipulating the surface facets and the potassium concentration, we hope to gain a deeper understanding of the K*_x_*W_7_O_22_ (K*_x_*WO)-based acetone sensing mechanism, and further improve the material property for its use in a breath acetone sensor for diagnosing diabetes and monitoring the disease.

## 2. Materials and Methods

K*_x_*WO is grown using the hydrothermal method [23]. A precursor solution containing Na_2_WO_4_ 2H_2_O (95%, Alfa Aesar, Tewksbury, MA, USA), oxalic acid dehydrate (>99%, VWR, Radnor, PA, USA), K_2_SO_4_ (>99%, VWR, Radnor, PA, USA), and hydrochloric acid (36%–38%, Aqua Solutions Inc., Deer Park, TX, USA) is made. This solution is then put into a 30-mL autoclave for synthesis, and heated at 225 °C for 24 h. Poly(ethylene glycol)-*block*-poly(propylene glycol)-*block*-poly(ethylene glycol) (designated PEG-PPG-PEG; Pluronic L-121, Sigma-Aldrich, St. Louis, MO, USA) was added to the precursor solution last and stirred for 10 min before beginning hydrothermal growth. Precursor solutions of varying K_2_SO_4_ concentrations to obtain the products K*_x_*W_7_O_22_ with *x* = 1.2, 1.5, 1.8, 2.0, and 2.2 were made for testing how potassium content affects sensitivity of the final sensing material.

X-ray photoelectron spectroscopy (XPS) was done using a Thermo Electron K-Alpha system (Thermo Fischer Scientific, Waltham, MA, USA). The samples were tested for chemical content to show the changing stoichiometric ratio of potassium.

X-ray diffraction (XRD) was done on a Bruker AXS D8 Discover (Bruker AXS Inc., Madison, WI, USA) to study an as-synthesized K*_x_*WO crystalline structure. Samples were made by blade coating a paste made from K*_x_*WO powder and ethanol on glass substrates. A diffraction pattern was gathered from a 2-Theta of 10° to 45°.

Raman spectra were taken using an Aramis Confocal Raman Imaging System with Horiba Jobin Yvon’s Raman Spectrometer (Horiba Jobin Yvon Inc., Piscataway, NJ, USA). Raman can be used to identify the ε-WO_3_ phase, and, thus, whether the ferroelectric phase is present or not in the sample [4].

The Fourier-transform infrared spectrometer (FT-IR) used in this study was a Thermo Scientific Nicolet 8700 FT-IR spectrometer (Thermo Fischer Scientific, Waltham, MA, USA). The FT-IR spectra was used to find if any surface functionalization changes could be observed when growing with the surfactant.

The acetone sensing performance has been done through a programmable chemiresistive gas sensor measurement system, which has been described elsewhere [16,24]. The acetone vapor is generated from OVG-4 (Owlstone Inc., Westport, CT, USA) based on the theory of permeation tube. The concentration of acetone can be precisely controlled from 0 to 5 ppm. Once the acetone is exposed to the K*_x_*WO film, a resistant change can be detected and recorded through an advanced circuit of a signal collecting system.

## 3. Results

### 3.1. Characterization

#### 3.1.1. X-ray Diffraction (XRD) and X-ray Photoelectron Spectroscopy (XPS)

**Figure 1:**
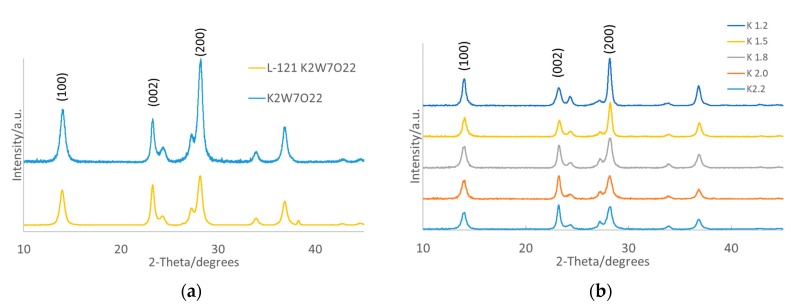
XRD of K*_x_*WO grown (**a**) with and without L-121 surfactant and (**b**) with *x* = 1.2, 1.5, 1.8, 2.0, and 2.2.

By utilizing high-resolution transmission electron microscopy (HRTEM) and XRD, previous work has shown that the crystal structure of KWO is similar to that of hexagonal tungsten trioxide (*h*-WO_3_) and Powder Diffraction File (PDF) card no. 85-2460. It can be tuned for higher acetone sensitivity by adjusting the growth temperature [14,16,25]. It has been reported that the controlled exposure of the crystal facets of WO_3_ have a high impact on acetone sensitivity and selectivity [14]. According to this information, we have modified K*_x_*WO in a similar fashion to adjust the crystal surfaces exposed, as shown in Figure 1a. 

The XRD of K*_x_*WO-L121 (e.g., here, KWO-L121) indicates that the introduction of the Pluronic L-121 surfactant has effectively changed the ratios of surface facets while still maintaining the same peak positions, which indicated the sample that maintained a hexagonal lattice structure. Therefore, to compare the samples, ratios of the (002) over the (200) peak heights were obtained. K_2.0_WO grown without surfactant shows a ratio of 0.426, and the addition of L-121 increases this ratio to 0.814. By utilizing a surfactant growth method, the facets have been altered enough to see if there will be a change in the materials sensing property, which will be discussed further on. 

Figure 1b shows the XRD for the samples with varying stoichiometric ratios of potassium. The general chemical formula of K*_x_*W_7_O_22_, where *x* was equal to 1.2, 1.5, 1.8, 2.0, and 2.2 for varying samples, was used. The first thing that should be noted is similar to growing with a surfactant. The potassium level directly controls the ratio of crystal facets. It can be seen that the (200) peak sharply increases at a K value of 1.5 and lower, while the (002) peak becomes significantly larger at a K value of 1.8 and above. Overall, we can see that, with a lower concentration of K, the (002) peak decreases and the (200) peak increases, and vice versa for increasing potassium concentration.

The data gathered by XRD indicated that we are able to control the crystal facets of the K*_x_*WO material via two pathways: addition of chemical additives during growth such as surfactants, and the adjustment of the concentration of potassium in K*_x_*WO.

**Figure A1:**
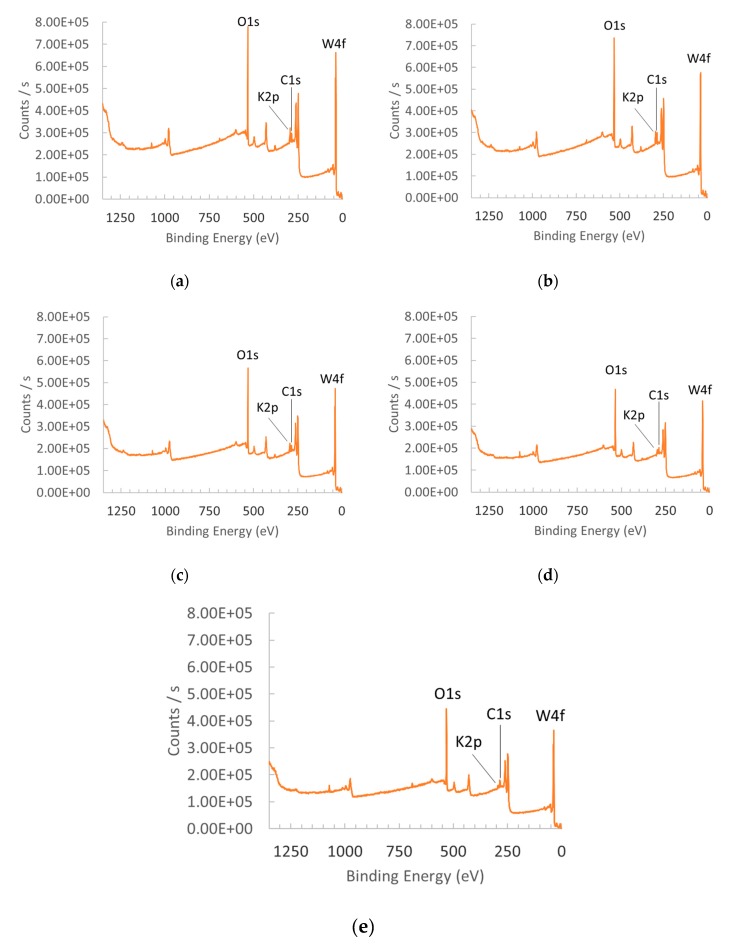
XPS of K*_x_*W_7_O_22_ with *x* = (**a**) 2.2, (**b**) 2.0, (**c**) 1.8, (**d**) 1.5, and (**e**) 1.2.

XPS was performed to show all samples did have the stoichiometric ratios intended. Table 1 shows the atomic percentages of K, W, and O observed from each sample. It should be noted that each sample had carbon in quantities of 14%–16% showing up as well as higher oxygen readings due to the presence of CO_2_ while collecting the sample data. With this in mind, we can see a clear trend of increasing K from samples K_1.2_WO to K_2.2_WO. This shows that we have, in fact, altered the stoichiometric ratio of K in the material by simply controlling the amount of K_2_SO_4_ used during synthesis. The XPS data can be found in Figure A1.

#### 3.1.2. Raman Spectroscopy

**Figure 2:**
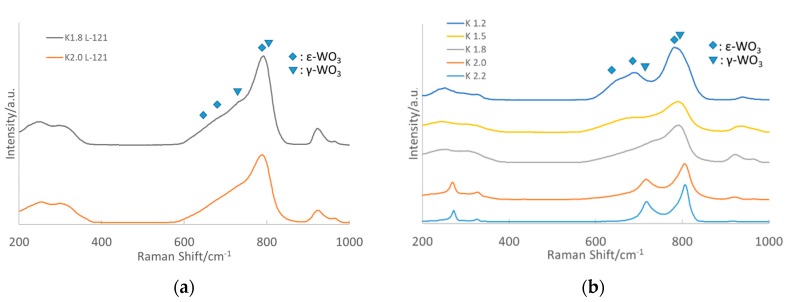
Raman of (**a**) K*_x_*WO with *x* = 1.8 and 2.0 grown with L-121 surfactant, and (**b**) K*_x_*WO with *x* = 1.2, 1.5, 1.8, 2.0, and 2.2.

Figure 2 shows the Raman obtained of different K*_x_*WO samples grown (a) with L-121, and (b) with a change in potassium content. Comparing K_1.8_WO, and K_2.0_WO when grown with L-121, we see little difference other than a more prominent ferroelectric phase observed by farther shouldering, which was previously reported by our group [25]. In detail, there are two primary peaks at 715 cm^−1^ and 805 cm^−1^ in Raman spectra, which indicate the presence of the γ-phase. With the addition of L-121, as shown in Figure 2a, a decrease of peaks related to the γ-phase was seen in K_1.8_WO-L121 and K_2.0_WO-L121. Instead, one primary peak below 800 cm^−1^ was present, which indicates a further shift to the ε-phase [15,25]. This result combined with the XRD data indicate KWO grown with L-121 has a stronger ferroelectric phase as well as a higher ratio of the *(002)* facet.

We can also see in Figure 2b a clear shift from the γ-phase to the ε-phase of WO_3_ when potassium levels are decreased. This is similar to results found when doping with chromium where a decrease in doping yielded more ε-WO_3_ [15]. As such, it is critically important to find the right component ratio of K in K*_x_*WO in order to optimize K*_x_*WO sensing performance. This is because both the ferroelectric phase and the (002) surface facet play an important role in K*_x_*WO as an acetone sensing material. To elaborate, if the potassium content of K*_x_*WO is too high, we see a strong drop in the ferroelectric phase. While, if the potassium content is too low, we see less expression of the (002) facet. This lets us conclude that there must be a balance where the potassium levels express enough of each property to maximize the material’s sensitivity.

#### 3.1.3. Fourier-Transform Infrared Spectroscopy (FT-IR)

**Figure 3:**
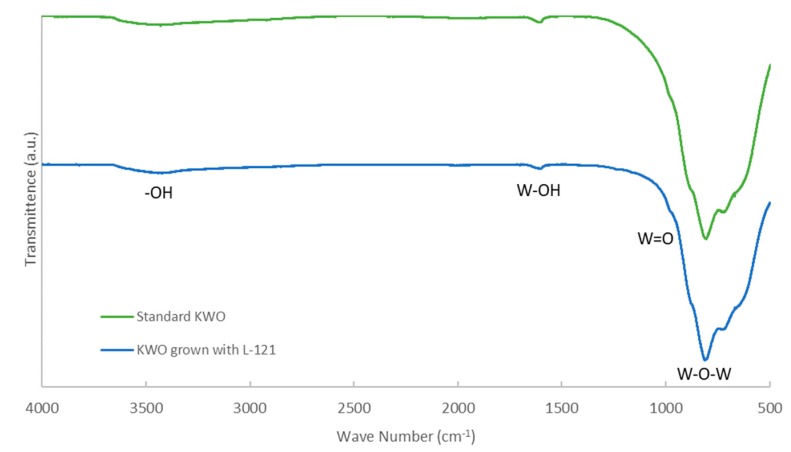
FT-IR of K*_x_*WO grown with and without L-121.

Figure 3 shows the FT-IR of the obtained samples grown with and without L-121. The peak at *ν* = 3410 cm^−1^ and weak peak at *ν* = 1590 cm^−1^ can be attributed to an -OH and an H_2_O stretching vibration. A strong band at *ν* = 806 cm^−1^ with shouldering at *ν* = 716 cm^−1^ corresponds to an O-W-O stretching vibration. In addition, weak shouldering at *ν* = 1030 cm^−1^ can be attributed to the W=O vibrational mode. A weak peak at *ν* = 1380 cm^−1^ is attributed to W-OH. Overall, we can conclude from this data that there is no significant change with the surface functionalization of the material. Therefore, any sensing changes that do occur are solely from the changing of the crystal facets and structure expressed.

### 3.2. Sensitivity Testing

**Figure 4:**
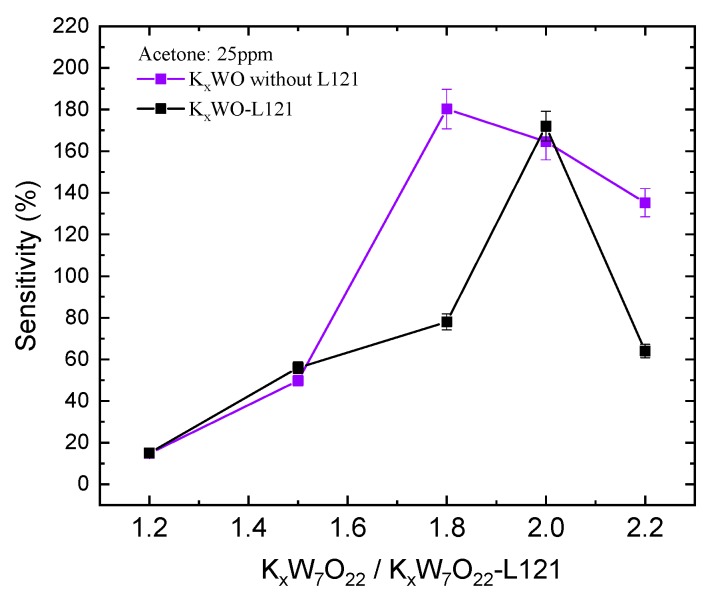
Sensing response to 25-ppm acetone using K*_x_*WO grown with and without L-121.

To examine the importance of potassium and L-121 in K*_x_*WO, sensing tests using sensor slides with different potassium concentrations has been conducted. The results are shown in Figure 4. This figure presents the sensitivity of 25 ppm acetone detection using K*_x_*WO samples made with *x* = 1.2, 1.5, 1.8, 2.0, and 2.2 as well as sensor slides made with K*_x_*WO that was treated for surface facet modification using L-121. Relative humidity (RH) also largely affects the sensing performance of a metal oxide semiconductor. The effect of humidity on KWO’s performance was reported previously, which showed that it can perform as an acetone sensor even in high humidity environments [26].

The sensitivities in Figure 4 were calculated from the sensor signal responses collected from multiple samples with varying potassium content using Equation (1). This is the standard equation used to study a chemiresistive gas-sensor’s sensitivity with *R_o_* being the initial resistance before exposure to acetone and *R* being the resistance after exposure to acetone. The *R_o_* value of KWO is generally in the low MΩ region and can increase to well above 100 MΩ when exposed to acetone. As observed, the best sensing performance needs to balance the concentration of potassium in K*_x_*WO and surfactant L-121 treatment. Looking back at Figure 1 and Figure 2, we see that this would make the most sense. This is because the (002) peak is relatively stronger compared to other peaks, and the presence of ε-WO_3_, the ferroelectric phase, is more prominent in the sample of K_2.0_WO-L121. This result confirms that both the surface facet (002) and ferroelectric phase play an important role in K*_x_*WO sensing to acetone.

[(*R* − *R_o_*)/*R_o_*] × 100% = sensitivity, (1)

**Figure 5:**
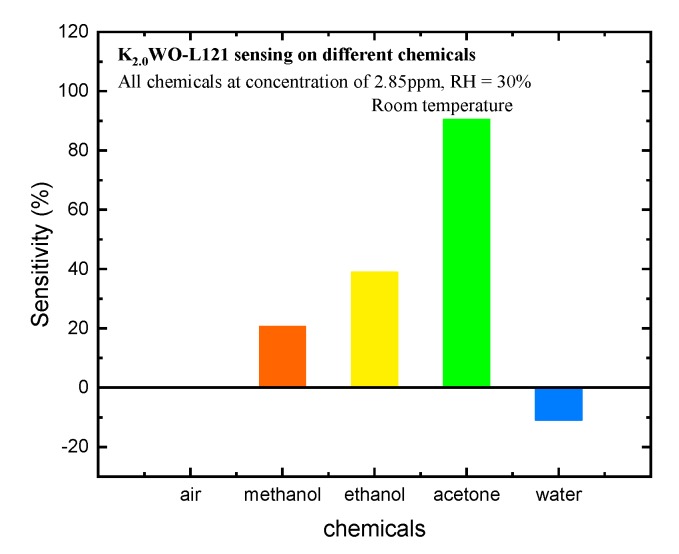
Selectivity testing of K_2.0_WO-L121 to 2.85 ppm of acetone, ethanol, methanol, and water vapor (the concentration of air is arbitrary).

Another important factor of a sensor’s performance is the selectivity. Figure 5 is the selectivity testing on 2.85 ppm of different chemicals such as acetone, ethanol, methanol, and water vapor for K_2.0_WO-L121. This indicated that K*_x_*WO with a potassium level of 2.0 treated with L-121 shows that the interaction with acetone has the highest sensitivity and selectivity. By manipulating the crystal facets of the K_x_WO material, we found that the (002) peak does have an important role for sensing acetone. Although the detailed explanation is still under investigation at current stages, we can briefly theorize that a higher number of coordinated unsaturated O atoms on the surface of the (002) facet than the (100) or (200) facet could be one factor. These O atoms allow for an easier interaction, which leads to larger charge transfer from the gas to the material surface. In addition, it has been reported that the O atoms on the (002) surface facet are asymmetrically arranged, which leads to a non-uniform distribution of the electron cloud-surface causing ferroelectricity [14]. This asymmetrical electron cloud makes the (002) facet form a local electric polarization, which allows for a dipole-dipole interaction between acetone and the surface of K*_x_*WO. Considering that acetone is a chemical with a higher dipole moment than ethanol, methanol, and water, these results are an example of the good selectivity of K*_x_*WO-L121 as an acetone-sensing material.

In a word, the manipulation of stoichiometric potassium levels as well as the introduction of L-121 during hydrothermal growth can improve K*_x_*WO sensing properties to detect acetone for a great potential application in non-invasive diabetes diagnosis and monitoring.

## 4. Discussion

The ferroelectric property of K*_x_*WO is not the only property that is important for acetone sensing. This study has shown that the surface facet (002) expressed also has an important role in the material’s sensing properties. In a summary, it was first shown that utilizing a surfactant (L-121) during growth could influence the surface facets expressed of the final material, and it was proven to not be an effect with different functional groups via FT-IR. Second, we have shown that the concentration of potassium, *x* in K*_x_*WO, can directly affect expressed facets as well as the ferroelectric phase of the final product. On the other hand, our results revealed that a balance is needed between exposed surface facets and the ferroelectric property to optimize the K*_x_*WO sensing response to acetone. We have observed this balance to be a stoichiometric ratio of 2:7:22 of K, W, and O, respectively. In addition, the introduction of L-121 during hydrothermal growth can create a more selective material toward acetone detection. This would allow for much more reliable readings when it is applied to a device for diabetes diagnosis and monitoring. Work in the future should include finding other methods to maximize the ferroelectric property while still keeping acceptable exposed surface facets for acetone detection.

## Figures and Tables

**Table 1:** Atomic make up of K*_x_*WO obtained from XPS.

Sample	Atomic % K	Atomic % W	Atomic % O
K_1.2_WO	2.70	19.61	57.22
K_1.5_WO	2.99	19.27	60.31
K_1.8_WO	4.42	19.69	59.47
K_2.0_WO	4.99	18.44	57.61
K_2.2_WO	5.48	18.57	59.54

## References

[1] Buszewski B., Kesy M., Ligor T., Amann A. (2007). Human exhaled air analytics: Biomarkers of diseases. Biomed. Chromatogr..

[2] Konvalina G., Haick H. (2014). Sensors for Breath Testing: From Nanomaterials to Comprehensive Disease Detection. Acc. Chem. Res..

[3] Deng C.H., Zhang J., Yu X.F., Zhang W., Zhang X.M. (2004). Determination of acetone in human breath by gas chromatography-mass spectrometry and solid-phase microextraction with on-fiber derivatization. J. Chromatogr. B-Anal. Technol. Biomed. Life Sci..

[4] Staerz A., Weimar U., Barsan N. (2016). Understanding the Potential of WO_3_ Based Sensors for Breath Analysis. Sensors.

[5] Tang B.L., Jiang G.H., Chen W.X., Wan J.M. (2015). First-Principles Study on Hexagonal WO_3_ for HCHO Gas Sensing Application. Acta Metall. Sin. Engl. Lett..

[6] Supothina S., Suwan M., Wisitsoraat A. (2014). Hydrothermal synthesis of K_2_W_4_O_13_ nanowire with high H2S gas sensitivity. Microelectron. Eng..

[7] Righettoni M., Tricoli A., Pratsinis S.E. (2010). Si:WO_3_ Sensors for Highly Selective Detection of Acetone for Easy Diagnosis of Diabetes by Breath Analysis. Anal. Chem..

[8] Barsan N., Weimar U. (2001). Conduction model of metal oxide gas sensors. J. Electroceramics.

[9] Gurlo A., Riedel R. (2007). In situ and operando spectroscopy for assessing mechanisms of gas sensing. Angew. Chem. Int. Ed..

[10] Morrison S. (1987). Mechanism of semiconductor gas sensor operation. Sens. Actuators.

[11] Ma L., Ma S.Y., Shen X.F., Wang T.T., Jiang X.H., Chen Q., Qiang Z., Yang H.M., Chen H. (2018). PrFeO_3_ hollow nanofibers as a highly efficient gas sensor for acetone detection. Sens. Actuators B-Chem..

[12] Wang X.F., Ma W., Jiang F., Cao E.S., Sun K.M., Cheng L., Song X.Z. (2018). Prussian Blue analogue derived porous NiFe_2_O_4_ nanocubes for low-concentration acetone sensing at low working temperature. Chem. Eng. J..

[13] Shin J., Choi S.J., Lee I., Youn D.Y., Park C.O., Lee J.H., Tuller H.L., Kim I.D. (2013). Thin-Wall Assembled SnO_2_ Fibers Functionalized by Catalytic Pt Nanoparticles and their Superior Exhaled-Breath-Sensing Properties for the Diagnosis of Diabetes. Adv. Funct. Mater..

[14] Jia Q.Q., Ji H.M., Wang D.H., Bai X., Sun X.H., Jin Z.G. (2014). Exposed facets induced enhanced acetone selective sensing property of nanostructured tungsten oxide. J. Mater. Chem. A.

[15] Wang L., Teleki A., Pratsinis S.E., Gouma P.I. (2008). Ferroelectric WO(3) nanoparticles for acetone selective detection. Chem. Mater..

[16] Wang D.L., Zhang Q., Hossain M.R., Johnson M. (2018). High Sensitive Breath Sensor Based on Nanostructured K_2_W_7_O_22_ for Detection of Type 1 Diabetes. IEEE Sens. J..

[17] Pokhrel S., Simion C.E., Teodorescu V.S., Barsan N., Weimar U. (2009). Synthesis, Mechanism, and Gas-Sensing Application of Surfactant Tailored Tungsten Oxide Nanostructures. Adv. Funct. Mater..

[18] Hu L.H., Peng Q., Li Y.D. (2008). Selective Synthesis of Co_3_O_4_ Nanocrystal with Different Shape and Crystal Plane Effect on Catalytic Property for Methane Combustion. J. Am. Chem. Soc..

[19] Han X.G., Li L., Wang C. (2012). Synthesis of Tin Dioxide Nanooctahedra with Exposed High-Index {332} Facets and Enhanced Selective Gas Sensing Properties. Chem. Asian J..

[20] Boppella R., Anjaneyulu K., Basak P., Manorama S.V. (2013). Facile Synthesis of Face Oriented ZnO Crystals: Tunable Polar Facets and Shape Induced Enhanced Photocatalytic Performance. J. Phys. Chem. C.

[21] Zhang H.B., Yao M.S., Bai L.Y., Xiang W.C., Jin H.C., Li J.L., Yuan F.L. (2013). Synthesis of uniform octahedral tungsten trioxide by RF induction thermal plasma and its application in gas sensing. Crystengcomm.

[22] Arai M., Hayashi S., Yamamoto K., Kim S.S. (1990). Raman Studies of Phase-transitions in Gas-evaporated WO_3_ Microcrystals. Solid State Commun..

[23] Yoshimura M., Byrappa K. (2008). Hydrothermal processing of materials: Past, present and future. J. Mater. Sci..

[24] Hossain M.R., Zhang Q.F., Johnson M., Wang D.L. (2018). Highly Sensitive Room-Temperature Sensor Based on Nanostructured K_2_W_7_O_22_ for Application in the Non-Invasive Diagnosis of Diabetes. Sensors.

[25] Johnson M., Zhang Q., Wang D.L. (2019). Room-Temperature Ferroelectric K_2_W_7_O_22_ (KWO) Nanorods as a Sensor for Detection of Acetone. Med Devices Sens..

[26] Hossain M.R., Zhang Q.F., Johnson M., Wang D.L. (2017). Investigation of humidity cross-interference effect on acetone breath sensor based on nanostructured K_2_W_7_O_22_. Eng Press.

